# Analyses of Endothelial Cells and Endothelial Progenitor Cells Released Microvesicles by Using Microbead and Q-dot Based Nanoparticle Tracking Analysis

**DOI:** 10.1038/srep24679

**Published:** 2016-04-20

**Authors:** Jinju Wang, Yun Zhong, Xiaotang Ma, Xiang Xiao, Chuanfang Cheng, Yusen Chen, Ifeanyi Iwuchukwu, Kenneth J. Gaines, Bin Zhao, Shiming Liu, Jeffrey B. Travers, Ji C. Bihl, Yanfang Chen

**Affiliations:** 1 Department of Pharmacology and Toxicology, Boonshoft School of Medicine, Wright State University, Dayton, Ohio, 45435, USA; 2 Department of Cardiology, Guangzhou Institute of Cardiovascular Disease, the Second Hospital of Guangzhou Medical University, Guangzhou, 510000, China; 3 Guangdong Key Laboratory of Age-Related Cardiac and Cerebral Diseases, Institute of Neurology, Affiliated Hospital of Guangdong Medical College, Zhanjiang, 524000, China; 4 Department of Neurology, Ochsner Medical Center, Jefferson, LA, 70121, USA; 5 Departments of Neurology and Internal Medicine, Boonshoft School of Medicine, Wright State University, Dayton, Ohio, 45435, USA; 6 Dayton VA Medical Center, Dayton, Ohio, 45428, USA

## Abstract

Accurate analysis of specific microvesicles (MVs) from biofluids is critical and challenging. Here we described novel methods to purify and detect MVs shed from endothelial cells (ECs) and endothelial progenitor cells (EPCs) by combining microbeads with fluorescence quantum dots (Q-dots) coupled nanoparticle tracking analysis (NTA). In the *in vitro* screening systems, we demonstrated that 1) anti-CD105 (EC marker) and anti-CD34 (EPC marker) conjugated-microbeads had the highest sensitivity and specificity for isolating respective MVs, which were confirmed with negative controls, CD41 and CD235a; 2) anti-CD144 (EC marker) and anti-KDR (EPC marker) conjugated-Q-dots exhibited the best sensitivity and specificity for their respective MV NTA detection, which were confirmed with positive control, anti-Annexin V (MV universal marker). The methods were further validated by their ability to efficiently recover the known amount of EC-MVs and EPC-MVs from particle-depleted plasma, and to detect the dynamical changes of plasma MVs in ischemic stroke patients, as compared with traditional flow cytometry. These novel methods provide ideal approaches for functional analysis and biomarker discovery of ECs- and EPCs- derived MVs.

Microvesicles (MVs, diameter: 120–1000 nm) are nanoscale extracellular vesicles budding from cellular membrane^1^,^2^,^3^,^4^,^5^. Upon released, MVs enter into the adjacent extracellular space and could travel to remote places to exert a physiological activity. Accumulating evidence has revealed that MVs mediate intercellular physiological communication and participate in the pathological process of diseases including cardiovascular diseases, cancer, and autoimmune diseases^6^,^7^. MVs can also be released into biological fluids such as plasma, urine, cerebrospinal fluid, which enable them to serve as potential novel biomarkers for diseases^8^,^9^.

Endothelial cells (ECs) play an important role in the development of cardiovascular diseases and can release MVs in response to activation or apoptosis^10^. The level of circulating EC-released MVs (defined as CD105^+^CD144^+^ cEC-microparticles) has been shown to be increased in ischemic stroke patients^11^. Likewise, our group has demonstrated that the level of circulating EC-MVs (cEC-MVs) is increased and positively correlated with the ischemic damage in db/db diabetic mouse^12^. Circulating endothelial progenitor cells (cEPCs) are adult stem cells generated from bone marrow. EPCs promote endothelial repair through replacing defective or injured mature ECs in vascular diseases^13^. An earlier clinical study reported that high cEPC-MVs (defined as CD34^+^KDR^+^ cEPC-microparticles) level was a predictor of aortic stiffness in cardiovascular diseases^13^. Later on, we found that the level of cEPC-MVs was elevated in db/db diabetic mouse^12^. All of these findings indicate that cEC-MVs and cEPC-MVs could serve as new surrogate biomarkers of vascular diseases. Nevertheless, it should be noted that flow cytometry has been used to detect EC-MVs and EPC-MVs in these studies. Since flow cytometry has analytic limitation in detecting MVs with size less than 300 nm^14^,^15^, sensitive and specific methods for isolation and detection of specific MVs are needed, especially for their functional study and biomarker discovery.

Microbeads are often used to isolate and enrich specific cell subpopulations via their coated antibodies against specific surface antigens of cells^16^. Since MVs carry the antigens of their parent cells^12^,^17^, specific antibody-conjugated microbeads can be used to isolate and purify MVs from the mixtures of extracellular vesicles. As showed by others and us, nanoparticle tracking analysis (NTA) can detect vesicles as small as 30 nm in diameter^18^,^19^,^20^ and count specific MVs using antibodies conjugated to fluorescent quantum dots (Q-dots)^21^,^22^. A previous study has applied this strategy to isolate specific circulating syncytiotrophoblast derived-MVs from human placental perfusate^22^. These observations suggest that cell specific antibody conjugated-microbeads combining with fluorescence Q-dots and NTA could be able to isolate and detect cell-specific MVs in the biofluids.

In this study, we aimed to determine whether combination of cell specific antibody (anti-CD105 or anti-CD34) conjugated microbeads for isolation and cell specific antibody (anti-CD144 or anti-KDR) conjugated Q-dots for NTA detection can offer highly sensitive and specific methods for detecting cEC-MVs and cEPC-MVs.

## Results

### Analyses of particle size and Annexin V marker expression of EC-MVs and EPC-MVs

**Figure 1:**
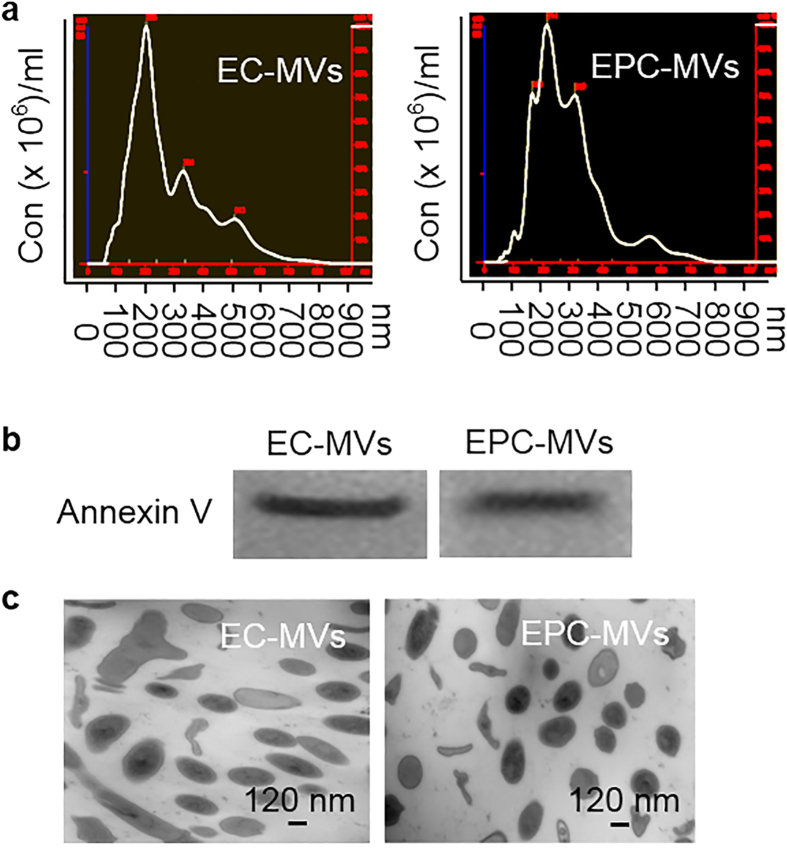
Characterization of EC-MVs and EPC-MVs by NTA, TEM and western blot. **(a**) representative NTA plots show size/concentration distribution of MVs isolated from cell cultures. (**b**) representative western blot bands showing specific expression of Annexin V in MVs. (**c**) TEM micrographs of MVs. EC-MVs: microvesicles released from endothelial cells. EPC-MVs: microvesicles released from endothelial progenitor cells. TEM: transmission electron microscopy. Annexin V: specific for MVs.

According to NTA and TEM analysis, the size of both EC-MVs and EPC-MVs ranged from 120–700 nm in diameter (Fig. 1a,b). The western blot results confirmed the expression of MV specific marker Annexin V in EC-MVs and EPC-MVs (Fig. 1c).

### The purification/detection efficiency and specificity of anti-CD105 and anti-CD34 conjugated-microbeads combining with Q-dots for isolating and detecting MVs from EC and EPC culture medium

In this study, we used EC-MVs and EPC-MVs released from cultured ECs and EPCs for establishing the methods. First of all, we tested the consistent of NTA for measuring MVs in series dilution by using 100 nm and 200 nm polystyrene beads and EC-MVs collected from the culture medium. As shown in Supplementary Fig. 1, the results of 100 nm and 200 nm polystyrene beads have demonstrated the good linear regression relationship between the expected concentration and the measured concentration of serial dilution (100 nm: R^2^ = 0.97; 200 nm: R^2^ = 0.98). Likewise, according to the results of the linear regression analysis, the measured EC-MV concentration was found to be proportionally decreased with serial dilution (R^2^ = 0.96). These data indicate that the concentration of EC-MVs detected by NTA was consistent with its series dilution.

**Figure 2:**
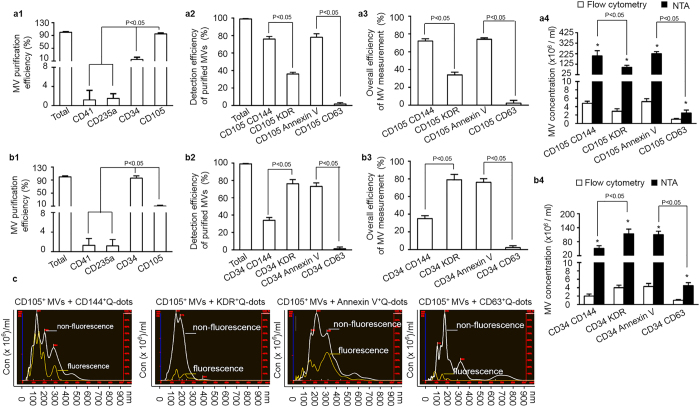
The efficiencies and specificities of the methods by combining microbeads with NTA for purifying and detecting EC-MVs and EPC-MVs. (**a1,b1**) the purification efficiencies and specificities of EC-MVs and EPC-MVs in the total MVs, which were collected from EC or EPC culture medium by ultra-centrifuge and isolated by various microbeads- conjugated antibodies against EC and EPC specific markers (CD105 and CD34 as well as negative controls, CD41, CD235a). (**a2,b2**) the detection efficiencies and specificities of EC-MVs in the total CD105^+^ MVs, or EPC-MVs in the total CD34^+^ MVs, that were labeled with CD144-, or KDR-, or Annexin V-, or CD63- conjugated Q-dots upon detected by fluorescence NTA. (**a3,b3**) the overall efficiency for measuring the CD105^+^ MVs or CD34^+^ MVs co-labeled with CD144-, or KDR-, or Annexin V-, or CD63- conjugated Q-dots in the total EC-MVs. (**a4,b4**) the absolute number of CD105^+^ MVs or CD34^+^ MVs that were positive for CD144, or KDR, or Annexin V, or CD63 in per ml EC or EPC culture medium. (**c**) representative plots showing the size/concentration distribution of the CD105^+^ beads isolated MVs under fluorescence/non-fluorescence modes. White curve: CD105^+^ MVs measured under light scatter (non-fluorescence) mode. Yellow curve: CD105^+^ Q-dots^+^ MVs measured under fluorescence mode. CD41: specific for platelets; CD235a: specific for erythrocytes; CD34: specific for endothelial progenitor cells; CD105: specific for endothelial cells; CD144: vascular endothelial antigen; KDR: EPC antigen; Annexin V: MV specific antigen; CD63: exosomal specific antigen. EC-MVs: microvesicles released from endothelial cells. EPC-MVs: microvesicles released from endothelial progenitor cells. N = 4/group.

As described, the specific microbeads captured MVs were analyzed by NTA under light scatter mode. The results showed that anti-CD105 conjugated-microbeads had the highest efficiency (>95%) for purifying EC-MVs (Fig. 2a1), and anti-CD34 conjugated-microbeads had the highest efficiency (>95%) for purifying EPC-MVs (Fig. 2b1), than anti-CD41 (specific for platelets) or anti-CD235a (specific for erythrocytes) conjugated microbeads did.

Since one specific surface antigen cannot accurately define these MVs are EC-MVs and EPC-MVs^10^,^12^, we incubated those microbeads captured MVs with the other cell-origin marker, CD144 (EC marker) or KDR (EPC marker). Results revealed that the detection rate of CD105^+^CD144^+^ EC-MVs was approximately 76% and of CD34^+^KDR^+^ EPC-MVs was approximately 73% (Fig. 2a2,b2). When taken isolation and detection together, the overall efficiencies of detecting CD105^+^CD144^+^ EC-MVs and CD34^+^KDR^+^ EPC-MVs were over 70% (Fig. 2a3,b3). In addition, the results showed that over 76% of CD105^+^ MVs and CD34^+^ MVs co-labeled with the MV universal marker Annexin V (a positive control) and only a small portion was positive for EX specific marker CD63 (a negative control), suggesting a very low amount of EXs in MV samples (Fig. 2a3,b3). When compared to the results from flow cytometery, the MV concentrations detected by NTA were almost two-orders of magnitude higher (Fig. 2a4,b4). Of note, analysis of the CD105^+^ MVs (white curve) and Q-dots labeled CD105^+^MVs (yellow curve) showed overlap size profiles (Fig. 2c), indicating that Q-dots binding did not change the physical characters of MVs.

All together, these data indicate that specific MVs could be specifically isolated from a particle pool and enumerated by using fluorescence NTA.

### Combining anti-CD105 or anti-CD34 conjugated-microbeads and Q-dots efficiently recovered and detected EC-MVs and EPC-MVs from particle-depleted plasma

First, we prepared particle-depleted plasma by series of centrifugation. We analyzed the size distribution of particles in the pelleted-MVs, MV-depleted plasma and particle-depleted plasma by using NTA. Our data showed that over 89% of particles in the pelleted-MVs pool were larger than 120 nm in diameter, approximately 90% of particles were less than 120 nm in diameter in MV-depleted plasma, and only few particles were detectable in the particle-depleted plasma sample (Supplementary Table 1 and Fig. 2).

**Figure 3:**
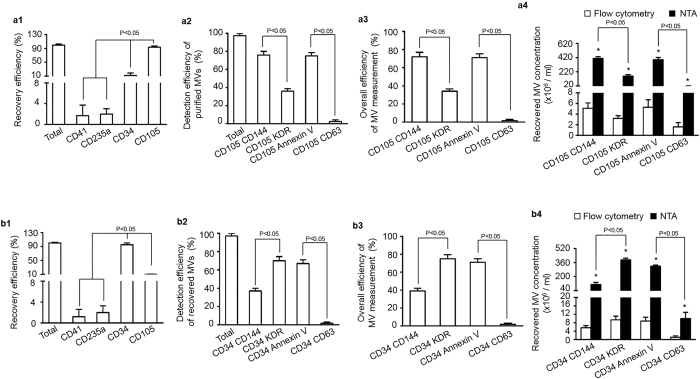
High recovery and detection efficiencies of MVs from particle-depleted plasma by using microbead purification and fluorescence NTA detection methods. (**a1,b1**) EC-MVs and EPC-MVs were recovered from particle-depleted plasma by using microbeads conjugated with various antibodies and analyzed by NTA. (**a2,b2**) the detection efficiency of recovered CD105^+^ MVs or CD34^+^ MVs that were labeled with secondary antibodies (CD144, or KDR, or Annexin V, or CD63) conjugated with Q-dots and analyzed by fluorescent NTA. (**a3,b3**) the overall efficiency for measuring the recovered CD105^+^ MVs or CD34^+^ MVs co-labeled with CD144-, or KDR-, or Annexin V-, or CD63- conjugated Q-dots. (**a4**,**b4**) the absolute number of recovered CD105^+^ MVs or CD34^+^ MVs that were positive for CD144, or KDR, or Annexin V, or CD63 in per ml EC or EPC culture medium. *p < 0.05, vs. flow cytometry. EC-MVs: microvesicles released from endothelial cells; EPC-MVs: microvesicles released from endothelial progenitor cells. N = 4/group.

In order to test the recovery efficiency of MVs from plasma, we added a known amount (6 × 10^8^ particles) of CD105^+^ MVs or CD34^+^ MVs into particle-depleted plasma and then assessed their respective recovery rate and detection efficiency using the above described methods. With the established methods, the known amounts of MVs (6 × 10^8^ particles) were added into particle-depleted plasma and followed by incubation with anti-CD105, anti-CD41, anti-CD235a or anti-CD34 conjugated-microbeads. NTA analysis showed that higher percentages (>95%) of EC-MVs were recovered by anti-CD105 or anti-CD34 conjugated-microbeads than that of anti-CD41 or anti-CD235a conjugated-microbeads did (Fig. 3a1,b1), further confirmed the reliability of the established isolation method. The fluorescence NTA results showed both the detection rates of CD105^+^CD144^+^ EC-MVs and of CD34^+^KDR^+^ EPC-MVs were over 70% (Fig. 3a2,b2). Again, take the recovery rate of isolation and detection together, the overall efficiencies of both types of MVs were approximately 70% (Fig. 3a3,b3). Similarly, both CD105^+^ MVs and CD34^+^ MVs were positively expressed Annexin V rather than CD63. These data further validated the high purification efficiency and specificity of the methods. As expected, the absolute number of CD105^+^CD144^+^ MVs or CD34^+^KDR^+^ MVs (Fig. 3a4,b4) were significantly higher as detected by fluorescence NTA than that detected by flow cytometry, suggesting the high sensitivity of NTA for detecting MVs and reflecting that the MV size is not affected by the biological complex of plasma.

### CEC-MVs and cEPC-MVs were isolated and enumerated by using anti-CD105/anti-CD144 or anti-CD34/anti-KDR and detected by fluorescence NTA

**Figure 4:**
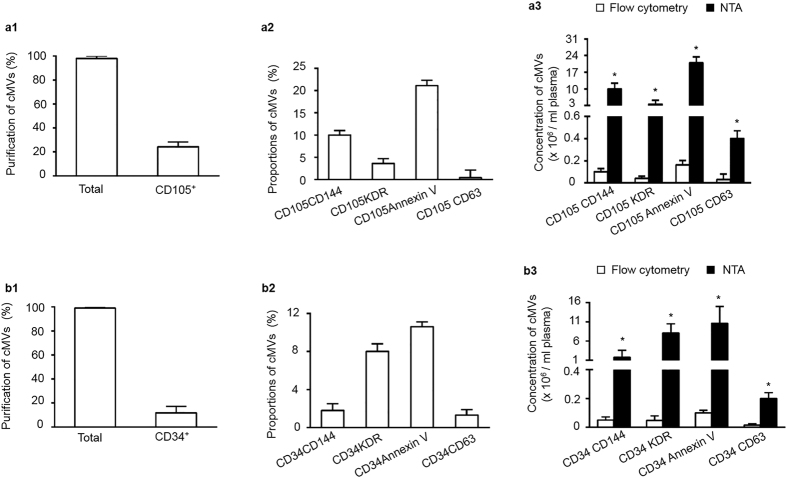
Identification of cEC-MVs and cEPC-MVs from human plasma by using anti-CD105 or anti-CD34 conjugated-microbeads and Q-dots combined with fluorescence NTA. (**a1,b1**) the proportions of CD105^+^ cMVs or CD34^+^ cMVs in plasma that were isolated by anti-CD105 or anti-CD34 conjugated-microbeads. (**a2,b2**) the proportion of CD105^+^ cMVs or CD34^+^ cMVs co-labeled with CD144-, or KDR-, or Annexin V-, or CD63- conjugated Q-dots. **(a3,b3**) The absolute number of CD105^+^ cMVs or CD34^+^ cMVs that were positive for CD144, or KDR, or Annexin V, or CD63 in per ml day 1 ischemic stroke patient plasma. *p < 0.05, vs. flow cytometry. N = 8/group. cEC-MVs: circulating microvesicles released from endothelial cells. cEPC-MVs: circulating microvesicles released from endothelial progenitor cells.

The results showed that approximately 24.3% of cMVs were captured by anti-CD105 conjugate-microbeads and 11.6% of cMVs was captured by anti-CD34 conjugated-microbeads, respectively (Fig. 4a1,b1). With the probe of Q-dots conjugated antibodies, about 10% of cMVs were CD105^+^CD144^+^ cEC-MVs, 20% of cMVs were CD105^+^Annexin V^+^ cEC-MVs, and a low portion of cMVs co-expressing CD105 and CD63, reflecting the low cross-contamination of exosomes in cMVs (Fig. 4a2). In the set samples for cEPC-MV analysis, the data showed that approximately 8% of total cMVs were CD34^+^KDR^+^ cMVs, 10.6% of total cMVs were CD34^+^Annexin V^+^ cMVs, and a low portion of cMVs co-expressing CD34 and CD63, also suggesting the low cross-contamination of exosomes in the isolated cMVs (Fig. 4b2). Of note, there was a significant difference in the concentration of different phenotype of cMVs as detected by NTA and flow cytometry, indicating the sensitivity of NTA. There were approximately 1.05 × 10^7^ CD105^+^CD144^+^ cEC-MVs and 8 × 10^6^ CD34^+^KDR^+^ cEPC-MVs in per ml plasma collected from day 1 after patient admission (Fig. 4a3,b3).

### Dynamic changes of cEC-MVs and cEPC-MVs in acute stroke patient plasma

**Figure 5:**
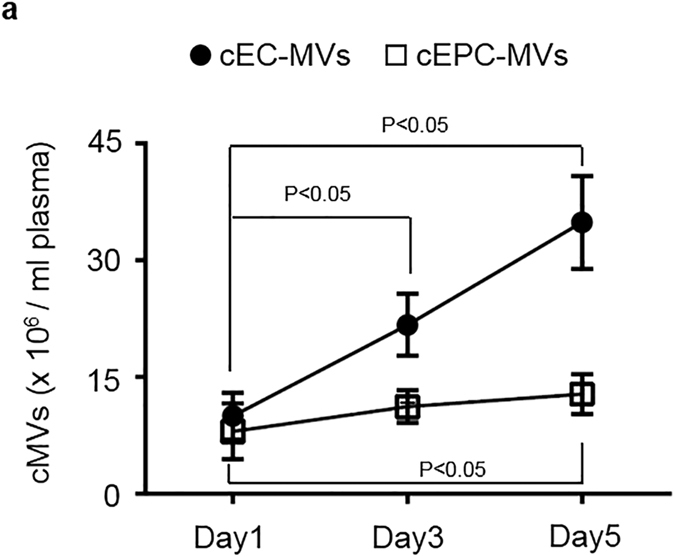
The dynamic change of cEC-MVs and cEPC-MVs in stroke patient plasma on days 1, 3 and 5 after admission. (**a**) the dynamic change of cEC-MVs and cEPC-MVs in per ml plasma on days 1, 3 and 5 after stroke patient admission upon analyzed by NTA. cEC-MVs: circulating microvesicles released from endothelial cells; cEPC-MVs: circulating microvesicles released from endothelial progenitor cells.

We assessed the dynamic changes of CD105^+^CD144^+^ cEC-MVs and CD34^+^KDR^+^ cEPC-MVs in patients at days 1, 3 and 5 after admission. As shown in Fig. 5, there were significant elevated levels of CD105^+^CD144^+^ cEC-MVs on days 3 and 5 as compared with that in day 1, indicating that the levels of cEC-MVs could serve as indicators for endothelium damage under the ischemic condition. Meanwhile, the level of CD34^+^KDR^+^ cEPC-MVs on day 5 was elevated as compared to that on day 1, but there was no difference between days 1 and 3.

## Discussion

MVs carry the characters of their parent cells and can mediate cell-to-cell communication, which enable them great clinical potentials for biomarker discovery and therapeutic target development^7^,^23^,^24^. MVs are released into extracellular spaces as an integral part of the intercellular microenvironment, which might play an important role in tissue/organ physiology and participate in the pathophysiology of disease as well^23^,^25^,^26^. Currently, accurate and sensitive methods for identification and quantification of specific MVs remain a roadblock in the research of this field. CEC-MVs and cEPC-MVs are two important types of MVs which could participate in the pathophysiological processes of cardiovascular diseases such as stroke, myocardial infarction and diabetes^11^,^12^. In this study, we have demonstrated the novel methods for isolating and detecting EC-MVs and EPC-MVs from cell culture medium and human plasma by combining microbead and Q-dot fluorescence NTA techniques.

In the *in vitro* cell culture systems, we developed the methods by using EC-MVs and EPC-MVs released from cultured ECs and EPCs. NTA analysis have demonstrated that EC-MVs and EPC-MVs collected from culture medium have a diameter of 120–1000 nm, which were in accordance with previous reports^1^,^2^. Others and us have shown that EC-microparticles express CD105 and/or CD144 of ECs^10^,^27^,^28^, and EPC-microparticles express CD34 and KDR of EPCs^12^,^27^. To examine whether these markers can be applied to capture the MVs from the culture medium of ECs and EPCs, we conjugated the anti-CD105 and anti-CD34 antibodies with magnetic microbeads to isolate respective MVs from the culture medium. The specificity of antibody-conjugated microbeads for isolating MVs was confirmed with negative controls. Of the antibody-conjugated microbeads studied, anti-CD105 conjugated microbeads captured the majority (>95%) of MVs collected from the EC culture medium, and only few MVs bind to the negative control antibodies CD41 (1.2%), CD235a (1.5%), or CD34 (15%). Similarly, the anti-CD34 conjugated microbeads had the highest degree of sensitivity in purifying MVs from EPC-MVs culture medium than CD41 or CD235a or CD105 did. These data reflect the high specificity and sensitivity of CD105 for isolating EC-MVs, and CD34 for isolating EPC-MVs. Since one surface antigen (CD105 or CD34) expression cannot define EC-MVs or EPC-MVs specifically, we applied the surface antigens (CD144 and KDR) conjugated to Q-dots to simultaneously label the microbeads captured MVs^10^,^12^,^27^. Of the antibody-conjugated Q-dots studied, the detection rates of CD105^+^CD144^+^ EC-MVs and CD34^+^KDR^+^ EPC-MVs were over 70%, respectively. Meanwhile, there was a low cross-reaction rate of these antibodies. All of these results demonstrate the specificity and sensitivity of the detection methods. When taking isolation and detection together, the overall efficiencies of CD105^+^CD144^+^ EC-MVs and CD34^+^KDR^+^ EPC-MVs were above 70%. In addition, the high overall efficiencies of CD105^+^Annexin V^+^ MVs (approximately 74%) and CD34^+^Annexin V^+^ MVs (approximately 75%), as well as the low overall efficiencies of CD105^+^CD63^+^ MVs (approximately 2.1%) and CD34^+^CD63^+^ MVs (approximately 2.3%) further validated the specificity and sensitivity of our methods. Flow cytometry has been widely used to characterize MVs in many studies^11^,^12^,^13^. However, due to its size detection limitation (minimal detection size is 300 nm), conventional flow cytometry may detect only a minority of all cell-derived MVs^29^,^30^. In this study, our results showed that the concentrations detected by NTA are two-order of magnitude higher than that detected by conventional flow cytometry. The latest generation of dedicated flow cytometer has been shown to be able to detect small microparticles^31^. Nevertheless, the forward scatter signals of flow cytometry are influenced by both the particle size and the refractive index which might lead to an underestimation of the particle size by using flow cytometry. In contrast, NTA determines particle size from Brownian motion which is independent of the refractive index of the particles^21^. Taken together, all of these data have demonstrated that specific antibody conjugated-microbeads combing with Q-dots can sensitively and specifically isolate and detect EC-MVs and EPC-MVs from culture medium.

In the present study, we further validated the methods by determining the recovery rate of isolation, detection rate, and the overall efficiency of MVs added into the MV-depleted plasma. The anti-CD105- and anti-CD34- conjugated microbeads isolated over 95% of the added EC-MVs and EPC-MVs from plasma, reflecting the reliability of the isolation method. After probed with anti-CD144 or anti-KDR-conjugated Q-dots, the detection rates of CD105^+^CD144^+^ EC-MVs and CD34^+^KDR^+^ EPC-MVs were over 72% upon the results obtained from NTA. The overall efficiencies of CD105^+^CD144^+^ EC-MV and CD34^+^KDR^+^ EPC-MV isolation and detection were approximately 70%, which are consistent with the data we obtained for developing the methods in the culture systems. Meanwhile, we also compared the sensitivities of our methods to that of the flow cytometer methods. The data revealed that the number of CD105^+^CD144^+^ EC-MVs or CD34^+^KDR^+^ EPC-MVs was almost two-orders of magnitude higher enumerated by NTA than that counted by the flow cytometer, which suggest that the established methods were much sensitive than flow cytometer in detecting these nanoscale particles.

Furthermore, we tested whether the methods were able to efficiently isolate the specific populations, cEC-MVs and cEPC-MVs, from the plasmas of ischemic stroke patients. We showed that approximately 10% of cMVs were CD105^+^CD144^+^ EC-MVs, and 8% of cMVs were CD34^+^KDR^+^ EPC-MVs in the day 1 plasma samples. The concentration of EC-MVs was approximately 1.05 ×10^7^ and of EPC-MVs was 8.1 × 10^6^ in 1 ml plasma. When compared to a previous report with the same types of antibodies, we found that our methods can detect at least two-order of magnitude higher of cEC-MVs than that detected by flow cytometer^27^. These findings suggest that our methods are specific and sensitive for isolating and detecting EC-MVs from plasma, although the disease status of the participator’s in the two studies might not be at the same stage. These data also indicate that our methods are applicable to analyze specific MVs from a small volume of clinical samples. In addition, with the novel methodologies, we examined the levels of cEC-MVs and cEPC-MVs in patient on days 3 and 5 after admission. Our results showed an elevated level of cEC-MVs was detected from the patient plasma on days 3 and 5 after admission, suggesting the vascular endothelium injury in ischemic stroke. Besides, the levels of cEPC-MVs were found to be increased on day 3 and significantly elevated on day 5, indicating a greater degree of EPC fragmentation into MVs as we previously reported^12^.

In conclusion, the present study describes a more accurate and effective strategy for isolating and analyzing specific MVs from culture medium and human plasma. Notably, this method is not limited to ischemic stroke, but can readily be adapted to other systems, ranging from cardiovascular diseases to inflammatory to neoplastic disorders. Given its ease, wide applicability and high discovery potential, we believe this methodology could be an important addition to the technical repertoire for the qualitative and quantitative assessment of specific MVs, as well as biomarker discovery of diseases.

## Methods

### Preparation of MVs from cell culture medium

Human ECs (Cell Systems; Kirkland, WA) and human EPCs (Amsbio; Cambridge, MA) were cultured according to the manufactures’ instruction. ECs and EPCs were cultured with CSC medium (Cell systems) or EPC basal medium (Amsbio) for 24 hr, respectively. The conditioned cell culture medium was collected and centrifuged at 300 g, 15 min, and followed by 2000 g, 30 min to remove cell debris^6^. Then cell-free culture medium was centrifuged at 20,000 g, 70 min to pellet MVs which were resuspended with phosphate-buffered saline (PBS) filtered through 20 nm-filter (Whatman, Pittsburgh, PA), and aliquoted for NTA and flow cytometry analyses.

### Isolating of EC-MVs and EPC-MVs from EC and EPC culture medium by using anti-CD105 or anti-CD34 conjugated microbeads

The pelleted MVs were incubated with 10 μl of Biotin-conjugated anti-CD105, or anti-CD41, or anti-CD235a, or anti-CD34 antibody (Miltenyi Biotec) in a 100 μl reaction volume for 2 hr, followed by adding 10 μl of anti-Biotin microbeads (Miltenyi Biotec) for 15 min, respectively. Then the microbeads-labeled MVs from the total MV suspension was separated by using a DynaMag-2 magnet (Life technology). After an overnight magnet separation, the fluid was gently removed and the microbeads-bound MVs were resuspended with 100 μl particle-free PBS which was filtered through 20 nm membrane filter (Anotop 25, Whatman). The multisort release reagent (10 μl; Miltenyi Biotec) was added to each sample to cleave off the microbeads. After 10 min, each sample was brought a 250 μl final volume with filtered PBS, and placed on the magnet. On the next day, the MVs in the fluid were collected and considered as CD105^+^, or CD41^+^, or CD235a^+^, or CD34^+^ MVs. All isolated MVs were enumerated by using the NTA NS300 system (Malvern Instruments). The purification efficiency was calculated as: the number of microbead positive MVs divided by the total number of MVs.

### Immunofluorescence labeling of CD105^+^ MVs and CD34^+^ MVs with Q-dots

The isolated CD105^+^ or CD34^+^ MVs were incubated with another antibody against CD144, or KDR, or Annexin V, or CD63 (1:200 dilution; Santa Cruz Biotechnology) for 2 hr in a 100 μl reaction volume. Followed by incubation with rabbit anti-goat IgG conjugated with Q-dot® 655 (1:350 dilution; Life Technologies) for 90 min at RT. Filtered PBS was used to dilute antibodies (CD144, KDR, CD63) and rabbit anti-goat IgG conjugated with Q-dot® 655. PBS supplemented with 2 mM calcium chloride solution was filtered through 20 nm filter and was used to dilute Annexin V^27^. Then, filtered PBS was added to the MV suspension (incubated with CD144, or KDR, or CD63) to give a final volume of 700 μl, and PBS supplemented with 2 mM calcium solution was added to the Annexin V group to give a final volume of 700 μl. All samples were analyzed by NTA NS300 system (Malvern Instruments). The detection efficiency of the CD105^+^ MVs and CD34^+^ MVs double labeled with a second Q-dots-conjugated antibody was calculated as: (CD105^+^Q-dots^+^) MVs% = (the number of CD105^+^Q-dots^+^ MVs)/(the total number of CD105^+^MVs); (CD34^+^Q-dots^+^) MVs% = (the number of CD34^+^Q-dots^+^ MVs)/(the total number of CD34^+^ MVs). The overall efficiency of measurement of the CD105^+^ MVs and CD34^+^ MVs double labeled with a second Q-dots-conjugated antibody was calculated as: the purification efficiency (%) x the detection efficiency (%).

### Nanoparticle tracking analysis

The NanoSight NS300 with a 405 nm laser instrument (Malvern Instruments, United Kingdom) was used to detect MVs^21^. The NanoSight polystyrene latex calibration beads, 100 nm and 200 nm, were used to check the instrument performance. The camera level was maintained at 10 for light scatter mode and 16 for fluorescence scatter mode between samples. Light scatter mode of NTA used the camera filter 1 and fluorescence mode used the camera filter 2 with the long-pass 430 nm filter in place. Three videos of typically 30 seconds duration were taken, with a frame rate of 30 frames per second. Data was analyzed by NTA 3.0 software (Malvern Instruments).

### Flow cytometry

The collected MVs were suspended with PBS or PBS supplemented with 2 mM calcium chloride^27^, and incubated with 10 μl PE-conjugated anti-CD105 or anti-CD34, and FITC-conjugated anti-goat CD63, or Annexin V, or anti-goat CD144 or KDR antibody (Santa Cruz Biotechnology). Isotype matched (IgG) nonspecific antibodies were served as negative controls. After incubation, labeled MVs were analyzed using a BD Accuri C6 flow cytometer (Accuri Cytometer, Ann Arbor, MI). The vesicle gate was determined using calibration microspheres of 200 nm and 1 μm flow cytometry beads (Molecular Probes; Eugene, OR). The data was analyzed by CFlow Plus (Accuri software). The detection rate of EMVs and EPC-MVs in flow cytometry was calculated as: the number of PE^+^FITC^+^ MVs divided by the total number of MVs. The detection efficiency of MVs was the absolute counts of PE^+^FITC^+^ MVs in per ml conditioned culture medium.

### Protein and western blot assays

Proteins from EC-MVs and EPC-MVs were isolated with lysis buffer (Thermo Scientific, FL) containing protease inhibitor. Protein concentration assay was performed using a Bradford assay kit (Bio Rad Laboratories). For western blot analysis, proteins were separated by electrophoresis and transferred onto nitrocellulose membranes. The membranes were blocked by incubating with 5% dry milk for 1 hr, and probed with the primary antibody anti-Annexin V (1:250; Abcam) at 4 °C for overnight, followed by incubation with HRP-conjugated secondary antibody. Blots were developed with enhanced chemiluminescence developing solutions.

### TEM of EC-MVs and EPC-MVs

The CD105^+^ MV and CD34^+^ MVs were fixed with 2% glutaraldehyde and post-fixed with 1% osmium (electron microscopy science, Hatfield, PA), embedded with Spurr resin (Sigma, Louis, MO), and baked at 60 °C according to the manufacturer’s instruction and our previous study^32^. Ultrathin sections (60–80 nm) were prepared with MT7000, mounted on 300-mesh copper grids, and stained with uranyl acetate and lead citrate. All samples were examined with an EM 208 (Philips) transmission electron microscope at an accelerating voltage of 70 KV.

### Preparation of particle-depleted plasma

A previous study has revealed that viscosity inversely correlates with the sedimentation efficiency of MVs using centrifugation^33^. The viscosity of plasma is 1.65 and of culture medium is 1.1 which is close to PBS. Therefore, in order to increase the MV sediment efficiency and to get particle-depleted plasma, we diluted the plasma with filtered PBS as previously reported^29^. In brief, human plasma was diluted 3× with filtered PBS and centrifuged at 200 g for 20 min, and 20,000 g for 70 min to remove MVs (the pellets were defined as pelleted-MVs). The supernatant (considered as MV-depleted plasma) was ultracentrifugation at 169,000 g for 6 hr to remove all the small particles. The supernatant after ultracentrifugation was considered as particle-free plasma. The pelleted-MVs, MV-depleted plasma and particle-depleted plasma were analyzed by NTA under non-fluorescence model. The percentage of large particles (≥120 nm) was calculated by: the number of large particles/the total number of particles. The percentage of small particles (<120 nm) was calculated by: the number of small particles/the total number of particles.

### Recovery of EC-MVs and EPC-MVs from particle-depleted plasma

A known amounts (6 × 10^8^ particles) of EC-MVs or EPC-MVs were added into 1 ml of prepared particle-depleted plasma. The EC-MVs/particle-depleted plasma mixture or EPC-MVs/particle-depleted plasma mixture was centrifuged at 20,000 g for 70 min at 4 °C to pellet EC-MVs or EPC-MVs. The pellets were resuspended with filtered PBS or PBS supplemented with 2 mM calcium chloride, and recovered by anti-CD105, or anti-CD34, or anti-CD41, or anti-CD235 conjugated-microbeads, followed by incubation with anti-CD144, anti-Annexin V, anti-KDR, or anti-CD63 conjugated-Q-dots and subsequently analyzed by fluorescence NTA. The recovery rate was calculated as: the number of microbeads positive MVs divided by the total number of MVs. The detection efficiency of the recovered CD105^+^ MVs or CD34^+^ MVs double labeled with a second Q-dots-conjugated antibody was calculated as: (CD105^+^Q-dots^+^) MVs% = (the number of CD105^+^Q-dots^+^ MVs)/(the total number of CD105^+^ MVs); (CD34^+^Q-dots^+^) MVs% = (the number of CD34^+^Q-dots^+^ MVs)/(the total number of CD34^+^ MVs). The overall efficiency of measuring the recovered CD105^+^ MVs and CD34^+^ MVs double labeled with Q-dots-conjugated antibody was calculated as: the recovery rate (%) × the detection efficiency (%).

### Study subjects

This study recruited 16 ischemic stroke patients from the Department of Neurology at the Ochsner Medical Center. Peripheral blood (3 ml) was collected from ischemic stroke patients on admission day (day 1) after stroke occurs. 16 patient plasma were divided and used for cEC-MVs and cEPC-MVs analysis. Exclusion criteria of subjects for this study included any of the situations^1^: infectious disease in a previous month^2^; histories of autoimmune disorder, peripheral vascular disease or stroke^3^; transient ischemic attack, cerebral infarction and cerebral hemorrhage^4^; liver failure and acute or chronic kidney disease^5^; recent myocardiac disease in last 3 months^6^, medications for lipid control, inflammation suppression or immunosuppression, and^7^ history of cancer. All experiment protocols were approved by the Department of Neurology at the Ochsner Medical Center and IRB committee at Wright State University. The methods were carried out in accordance with the approved guidelines. Written informed consent was obtained from each participant prior to enrollment in the study.

### Preparation and analyses of cMVs from human plasma

Whole blood samples (3 ml) were drawn from ischemic stroke patients at admission day (day 1), days 3 and 5 after admission through 20-gauge needles into vacutainers containing 3.13% sodium citrate (BD Sciences). All blood samples were kept at RT with gentle agitation and proceeded within 2 hr. The whole blood samples were centrifuged at 400 g for 35 min at 4 °C to collect plasma, then 1 ml of plasma was centrifuged at 2000 g for 20 min to remove platelet. The platelet free plasma (PFP) was frozen in cryotube in aliquots (1 ml/tube) at −80 °C until use. For analysis, 1 ml frozen PFP was thaw on RT, diluted with PBS to make up to 3 ml, and centrifuged at 20,000 g for 70 min to pellet cMVs. The pelleted cMVs were resuspended with filtered PBS or PBS supplemented with 2 mM calcium chloride, and then isolated by anti-CD105 or anti-CD34 conjugated microbeads, and analyzed by NTA. The microbeads positive cMVs were probed with anti-CD144, anti-Annexin V, anti-KDR, or anti-CD63 conjugated-Q-dots and analyzed by fluorescence NTA. The proportion of cMVs using fluorescence NTA was calculated as: (CD105^+^Q-dots^+^) cMVs % = CD105^+^Q-dots^+^ cMVs/total cMVs; or (CD34^+^Q-dots^+^) cMVs % = CD34^+^Q-dots^+^ cMVs/total cMVs. The absolute number of cMVs was the absolute counts of CD105^+^Q-dots^+^ cMVs or CD34^+^Q-dots^+^ cMVs in per ml human plasma.

### Statistical analysis

Experimental data were expressed as the mean ± SEM, and were analyzed using one- or two-way analysis of variance (ANOVA). The correlations of MV numbers with their protein concentrations were analyzed using the Spearman’s rank correlation test (SPSS version 17.0; SPSS, Chicago, IL). Values of P < 0.05 were considered to be statistical significance.

## Additional Information

**How to cite this article**: Wang, J. *et al*. Analyses of Endothelial Cells and Endothelial Progenitor Cells Released Microvesicles by Using Microbead and Q-dot Based Nanoparticle Tracking Analysis. *Sci. Rep.* **6**, 24679; doi: 10.1038/srep24679 (2016).

## Supplementary Material

Supplementary Information
