# Supplementary material for: Analyses of Endothelial Cells and Endothelial Progenitor Cells Released Microvesicles by Using Microbead and Q-dot Based Nanoparticle Tracking Analysis
Source: Sci Rep. 2016 Apr 20;6:24679. doi: 10.1038/srep24679 (PMC4837394; doi:10.1038/srep24679)
Supplement: Supplementary Information [file srep24679-s1.pdf]

Analyses of Endothelial Cells and Endothelial Progenitor Cells Released Microvesicles by Using Microbead and Q-dot Based Nanoparticle Tracking Analysis

Jinju Wang[1,+], Yun Zhong [1, 2, +], Xiaotang Ma[3, +], Xiang Xiao[1], Chuanfang Cheng[2], Yusen Chen[3], Ifeanyi Iwuchukwu[4] , Kenneth J. Gaines[4, 5], Bin Zhao[3], Shiming Liu[2], Jeffrey B. Travers[1, 6], Ji C. Bihl[1, 3, *] , Yanfang Chen[1, 2, 3, 5, *]

[1] Department of Pharmacology and Toxicology, Boonshoft School of Medicine, Wright State University, Dayton, Ohio, USA, 45435

[2] Department of Cardiology, Guangzhou Institute of Cardiovascular Disease, the Second Hospital of Guangzhou Medical University, Guangzhou, China, 510000

[3] Guangdong Key Laboratory of Age-Related Cardiac and Cerebral Diseases, Institute of Neurology, Affiliated Hospital of Guangdong Medical College, Zhanjiang, China, 524000

[4] Department of Neurology, Ochsner Medical Center, Jefferson, LA, USA, 70121

[5] Departments of Neurology and Internal Medicine, Boonshoft School of Medicine, Wright State University, Dayton, Ohio, USA, 45435

[6] Dayton VA Medical Center, Dayton, Ohio, USA, 45428

[+]: Equal contributors; *: Corresponding authors

Yanfang Chen, M.D, Ph.D.
Phone: (937) 775-3265, Fax: (937) 775-7221; Email: yanfang.chen@wright.edu
Ji C. Bihl, M.D., Ph, D.
Phone: (937) 775-5243, Fax: (937) 775-7221; Email: ji.bihl@wright.edu

Fig 1

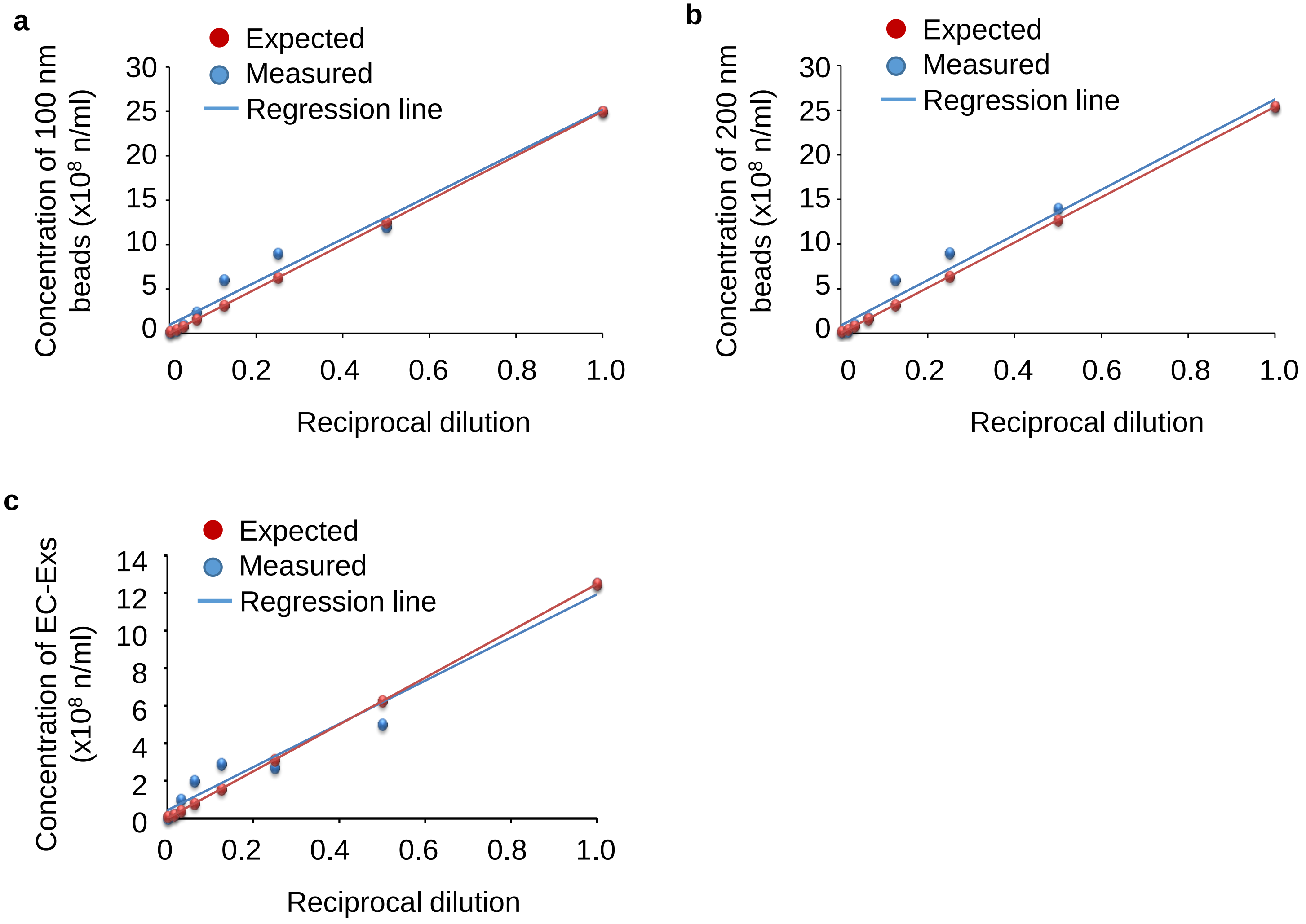

a
Expected
Measured
Regression line
Concentration of 100 nm beads (x10^8 n/ml)
30
25
20
15
10
5
0
0
0.2
0.4
0.6
0.8
1.0
Reciprocal dilution
b
Expected
Measured
Regression line
Concentration of 200 nm beads (x10^8 n/ml)
30
25
20
15
10
5
0
0
0.2
0.4
0.6
0.8
1.0
Reciprocal dilution
c
Expected
Measured
Regression line
Concentration of EC-Exs (x10^8 n/ml)
14
12
10
8
6
4
2
0
0
0.2
0.4
0.6
0.8
1.0
Reciprocal dilution

Fig 2

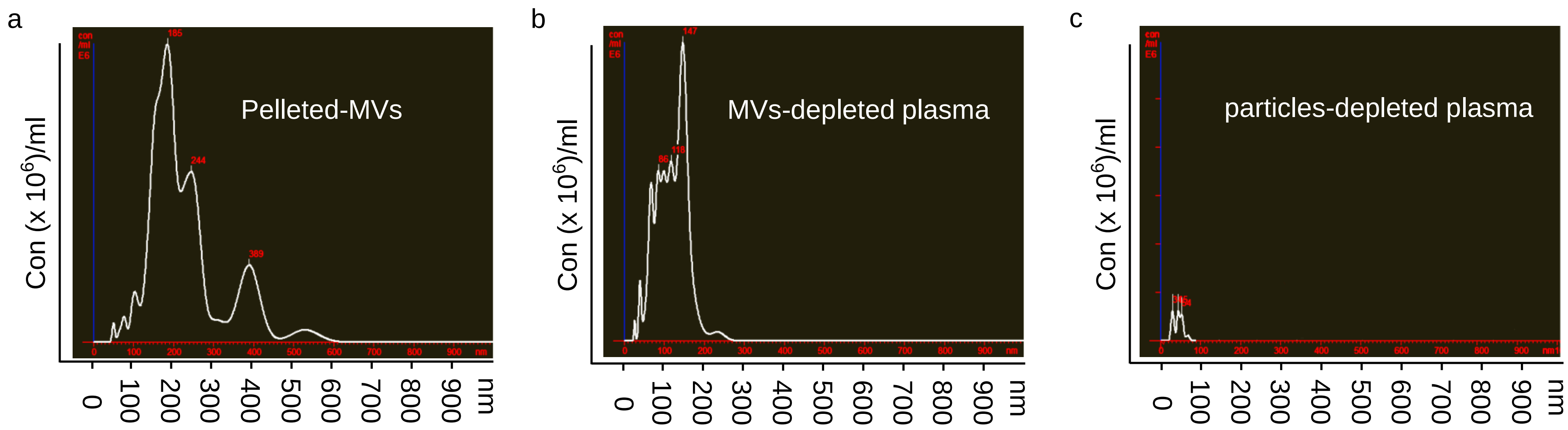

a
Pelleted-MVs
b
MVs-depleted plasma
c
particles-depleted plasma
Con (x 10⁶)/ml
0
100
200
300
400
500
600
700
800
900
nm

Table 1. Summarized data showing NTA analysis of particles from pelleted-MVs, MV-depleted plasma and particle-depleted plasma.

	% of total particles (< 120 nm)	% of total particles (≥120 nm)
Pelleted-MVs	11 ± 5	89 ± 5
MV-depleted plasma	90 ± 4	10 ± 4
particle-depleted plasma	1.2 ± 0.51	0.3 ± 0.2

Pelleted-MVs: MVs pelleted by centrifugation at 20,000 g for 70 min. MV-depleted plasma: the supernatant of plasma centrifuged at 20,000 g for 70 min. Particle-depleted plasma: the supernatant of plasma centrifuged at 169,000 g for 6 hr.

Supplementary methods

1. Beads and MV series dilution analysis by NTA.

To demonstrate the consistent of NTA for detecting different concentrations of MVs, we tested the instrument by measuring serial dilution (1:2, 1:4, 1:8, 1:16, 1:32, 1:64, 1:100) of 100 nm polystyrene beads and 200 nm polystyrene beads in filtered PBS. Similarly, EC-MVs collected from culture medium was serially diluted (1:2, 1:4, 1:8, 1:16, 1:32, 1:64, 1:100) with filtered PBS. The samples were mixed before introduction into the sample chamber and three videos typically 30 seconds duration were taken. NTA post-acquisition setting were kept constant between samples. Data was analyzed by NTA 3.0 software (Malvern Instruments).

Figure legend for supplementary data

Fig 1. Linear regression analysis of expected concentration and measured concentration of beads and MVs in serial dilutions. a-b, the plots showing the linear regression relationship between the expected concentrations and measured concentration at series dilutions of 100 nm and 200 nm polystyrene beads. c. the plot showing the linear regression relationship between the expected concentrations and measured concentration at series dilutions of EC-MVs collected from the culture medium. EC-MVs: microvesicles released from endothelial cells. N=4/group.

Fig 2. NTA analysis of particles in pelleted-MVs, MV-depleted plasma and particle-depleted plasma. A. representative NTA images showing the size distribution of particles in pelleted-MVs. B, representative NTA images showing the size distribution of particles in MV-depleted plasma. Pelleted-MVs: MVs pelleted by centrifugation at 20,000 g for 70 min. MV-depleted plasma: the supernatant of plasma centrifuged at 20,000 g for 70 min. Particle-depleted plasma: the supernatant of plasma centrifuged at 169,000 g for 6 hr.

Table 1. Summarized data showing NTA analysis of particles from pelleted-MVs, MV-depleted plasma and particle-depleted plasma.
